# Prognostic Value of Treatment-Related Body Composition Changes in Metastatic NSCLC Receiving Nivolumab

**DOI:** 10.3390/medicina62010098

**Published:** 2026-01-02

**Authors:** Erkam Kocaaslan, Ali Kaan Güren, Fırat Akagündüz, Ahmet Demirel, Mustafa Alperen Tunç, Burak Paçacı, Yeşim Ağyol, Pınar Erel, Abdüssamed Çelebi, Selver Işık, Ezgi Çoban, Nazım Can Demircan, Salih Özgüven, Zeynep Ceren Balaban Genç, Nargiz Majidova, Nadiye Sever, Murat Sarı, Osman Köstek, İbrahim Vedat Bayoğlu

**Affiliations:** 1 Department of Medical Oncology, Marmara University Pendik Training and Research Hospital, Istanbul 34899, Turkeyfratakagunduz0@gmail.com (F.A.); dr-selver83@hotmail.com (S.I.);; 2 Department of Nuclear Medicine, Marmara University Pendik Training and Research Hospital, Istanbul 34899, Turkey; 3 Department of Medical Oncology, VM Medical Park Maltepe Hospital, Istanbul 34843, Turkey; 4 Department of Medical Oncology, Haydarpaşa Numune Training and Research Hospital, Istanbul 34668, Turkey

**Keywords:** non-small cell lung cancer, nivolumab, sarcopenia, body composition, skeletal muscle loss, subcutaneous fat density, immunotherapy

## Abstract

*Background and Objectives:* This study aimed to evaluate the prognostic impact of baseline body composition measurements and changes in muscle and adipose tissue during treatment on overall survival (OS) in metastatic non-small cell lung cancer (NSCLC) patients treated with nivolumab. *Materials and Methods:* Eighty-eight metastatic NSCLC patients who were initiated on nivolumab between January 2022 and December 2024 were retrospectively analyzed. Body composition parameters were derived from baseline and 3-month ^18^F-FDG PET/CT scans at the L3 level, including psoas muscle index (PMI), skeletal muscle index (SMI), intramuscular adipose content (IMAC), and subcutaneous fat density (SFD). Treatment-related changes in body composition were evaluated, and survival analyses were performed using Kaplan–Meier estimates and Cox regression models. *Results:* Overall, 34.1% (*n* = 30) of patients were classified as sarcopenic. Median OS was significantly longer in non-sarcopenic patients (19 months vs. 5 months, *p* < 0.001). In univariate analysis, older age, higher comorbidity burden, liver metastasis, baseline sarcopenia, and adverse treatment-related changes in muscle and nutritional parameters were found to be associated with OS. In multivariate analysis, only unfavorable changes in skeletal muscle (ΔSMI; HR 3.39, *p* = 0.003) and subcutaneous fat radiodensity (ΔSFD; HR 2.45, *p* = 0.02) remained independent adverse prognostic factors. Baseline body composition parameters did not maintain their independence in multivariate models. *Conclusions:* Our study demonstrates that muscle loss or insufficient gain and unfavorable changes in subcutaneous fat radiodensity during nivolumab treatment more strongly predict overall survival compared to baseline measurements. These findings highlight the clinical importance of monitoring dynamic body composition throughout treatment, rather than static assessments, in NSCLC patients receiving immunotherapy.

## 1. Introduction

Lung cancer is one of the leading causes of cancer-related deaths worldwide, with approximately 2.5 million new cases and 1.8 million deaths reported in 2022 [1]. Non-small cell lung cancer (NSCLC) accounts for the majority of all lung cancers, and most patients are in the metastatic stage at diagnosis. While programmed cell death-1 (PD-1) inhibitors, particularly nivolumab, have provided significant progress in the treatment of metastatic NSCLC, only a limited number of patients achieve durable responses [2]. Therefore, identifying reliable biomarkers that can predict treatment response is a critical clinical need.

Body composition is closely related to immune response and metabolic processes and is an important prognostic factor in advanced cancer patients [3,4]. Sarcopenia has been associated with poor survival in NSCLC, as in many malignancies [5]. Objective measures of muscle mass, such as the psoas muscle index (PMI), skeletal muscle index (SMI), and IMAC, an indicator of intramuscular adiposity, are widely used in the quantitative assessment of sarcopenia [6,7].

In addition to skeletal muscle parameters, adipose-tissue features have gained attention as potential prognostic markers. Subcutaneous adiposity (e.g., subcutaneous fat area/amount) has been associated with survival outcomes in cancer patients [8]. More recently, CT-derived subcutaneous fat radiodensity (HU), which may reflect adipose tissue quality and inflammatory remodeling, has also been reported as prognostically relevant in several malignancies [9]. However, data on the prognostic role of subcutaneous fat radiodensity during immunotherapy for NSCLC remain limited. In contrast, in the context of immunotherapy, muscle loss—particularly when occuring during treatment— has been reported to be associated with poor survival, independent of baseline sarcopenia [10]. Most existing studies focus only on muscle or fat measurements at the beginning of treatment and do not evaluate dynamic changes.

In this study, we aimed to evaluate the impact of baseline sarcopenia, muscle, and adipose tissue parameters, and their changes during treatment, on overall survival in metastatic NSCLC patients receiving nivolumab. We hypothesized that, compared with pretreatment static measurements, muscle loss and unfavorable subcutaneous fat radiodensity remodeling during treatment would have stronger prognostic effects on survival. With this comprehensive analysis based on real-world data, we aimed to identify dynamic body composition markers that could contribute to risk stratification in patients receiving immunotherapy.

## 2. Materials and Methods

### 2.1. Study Design and Participants

This study is a single-center retrospective analysis conducted at Marmara University Faculty of Medicine Hospital. Patients diagnosed with advanced non-small cell lung cancer (NSCLC) and initiated on nivolumab treatment between January 2022 and December 2024 were included in the study. Patients with an ^18^F-FDG PET/CT scan within two months before treatment initiation and who had completed at least three months of follow-up after initiating immunotherapy were included in the study population. Patients with stable brain metastases (showing no radiological progression and considered clinically controlled) who had previously undergone surgery or thoracic radiotherapy were also eligible.

Demographic information such as age, gender, smoking status, body mass index, and clinical variables such as ECOG performance score, histopathological subtype, comorbid diseases, and the distribution and number of metastases were obtained from patient records. Molecular data (EGFR, ALK, ROS1, and PD-L1 status) were also examined. Pediatric age group, patients with an interval of more than two months between PET/CT and treatment initiation, patients with autoimmune diseases, patients with another concurrent malignancy, and patients with missing clinical or laboratory data were excluded from the study. Additionally, all patients positive for EGFR, ALK, or ROS1 were excluded from the analyses.

**Figure 1:**
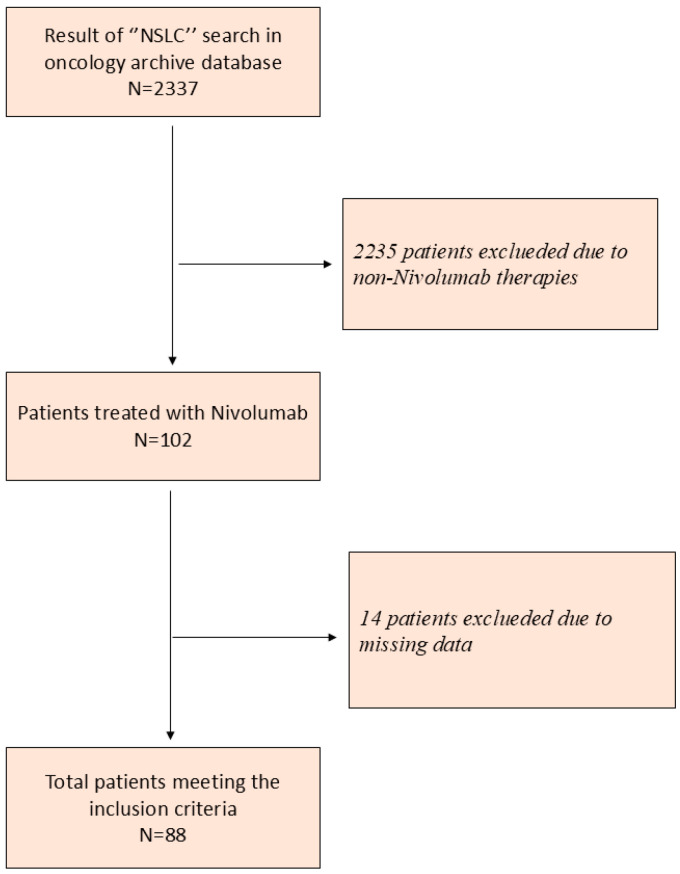
CONSORT flow diagram of patient selection.

A total of 2337 patients were identified through the “NSCLC” search performed in the oncology information system; 2235 were excluded because they received systemic therapies other than nivolumab. Among the remaining 102 patients, 14 with missing data were excluded, and ultimately, 88 patients were eligible for analysis (Figure 1). All patients received 3 mg/kg nivolumab as an intravenous infusion every two weeks. Treatment response was assessed using PERCIST (PET Response Criteria in Solid Tumors) criteria using PET/CT scans taken every three months from the start of treatment. Because treatment-related changes in body composition were assessed using PET/CT performed at 3 months, the study population effectively represents a 3-month landmark cohort, including patients who remained alive and on treatment at that time point.

The study was approved by the Clinical Research Ethics Committee of Marmara University Faculty of Medicine (Decision No: 09.2024.1475, 24 December 2024). Because all data were evaluated anonymously, written informed consent from the participants was not required. The study was conducted in accordance with the provisions of the Declaration of Helsinki and relevant legislation.

### 2.2. PET/CT Imaging Protocol

All examinations were performed on a Discovery ST PET/CT system (GE Healthcare, Milwaukee, WI, USA). Patients fasted for at least 6 h before imaging. A pre-injection blood glucose level of <126 mg/dL was required. Each patient received ^18^F FDG intravenously at a dose of 5 MBq/kg. After an uptake period of approximately 60 min, a low-dose, non-contrast CT scan was acquired from the upper thighs to the skull base using a 16-slice multi-detector scanner (GE Healthcare, Milwaukee, WI, USA; 80 mA, 140 kV, table speed 27 mm/rotation, slice thickness 3.3 mm) during shallow respiration. This was followed by a whole-body PET acquisition in 3D mode, with 3 min per bed position (six to eight positions), covering the same anatomical region. PET data were reconstructed using an iterative algorithm, and CT images were used for attenuation correction. Reconstructed PET images, with and without attenuation correction, were reviewed in axial, coronal, and sagittal planes on an Advantage Windows Workstation 4.5 using PET VCAR software.

### 2.3. Body Composition and Laboratory Measurements

All CT-based body composition measurements were performed on the CT component of the PET/CT examinations using the Advantage Windows Workstation (version 4.5; GE Healthcare, Milwaukee, WI, USA) with PET VCAR software. Segmentation and region-of-interest placement were performed manually using predefined Hounsfield unit thresholds, following a standardized protocol based on previously published methodology. All body composition measurements were performed by two experienced nuclear medicine physicians who were blinded to clinical outcomes and survival data, with consensus reached in cases of uncertainty. The third lumbar vertebra (L3) was selected as the anatomical landmark, defined as the axial level where both transverse processes were fully visible. For each patient, measurements were extracted from a single axial slice passing through L3 [11]. Based on prior studies, attenuation thresholds of −190 to −30 HU were used to define subcutaneous fat, and −29 to +150 HU were applied to identify skeletal muscle [2]. The average Hounsfield Unit (HU) was measured for both the psoas muscle and the total cross-sectional skeletal muscle area. For each patient, the bilaterally psoas muscle areas (mm^2^) and the total cross-sectional skeletal muscle area at the L3 vertebral level were quantified [12,13]. Subcutaneous adipose tissue was evaluated at the same L3 slice. To minimize heterogeneity and sampling bias, four spherical regions of interest (ROIs) (two anterior, two posterolateral) were placed symmetrically within the subcutaneous fat layer. For each ROI, the average HU was recorded, and the overall subcutaneous fat density (SFD, HU) was calculated as the arithmetic mean of all four values [9]. Body composition indices were normalized to height squared to minimize inter-individual variability. Accordingly, the psoas muscle index (PMI), skeletal muscle index (SMI) were calculated by dividing their respective cross-sectional areas (cm^2^) by the square of height (m^2^). Intramuscular adipose content (IMAC) was computed as the ratio of the mean HU of the multifidus muscle to the mean HU of subcutaneous fat, representing muscle quality independent of muscle size. Sarcopenia was defined as low skeletal muscle index (SMI): for women, SMI < 41 cm^2^/m^2^ regardless of BMI; and for men, SMI < 43 cm^2^/m^2^ when BMI < 25 kg/m^2^ or SMI < 53 cm^2^/m^2^ when BMI ≥ 25 kg/m^2^ [6]. Laboratory parameters were obtained from routine blood tests during the last 2 weeks before the start of treatment. The Prognostic Nutritional Index (PNI) was calculated using the standard formula: PNI = 10 × serum albumin (g/dL) + 0.005 × absolute lymphocyte count (/mm^3^). Changes in muscle-related parameters (PMI and SMI)and PNI were evaluated according to follow-up PET/CT and laboratory results performed at 3 months. Change rates were calculated as percentage change using the formula (3-month value—baseline value)/baseline value × 100 and expressed as ΔPMI, ΔSMI, and ΔPNI. In contrast, changes in subcutaneous fat density (ΔSFD) were calculated as the difference in Hounsfield units (HU) between the 3-month and baseline measurements (3-month value minus baseline value), because radiodensity is a linear parameter and percentage change may be misleading for HU-based measures with negative values. Subcutaneous fat density (SFD) was expressed in Hounsfield units, with higher (less negative) HU values indicating increased radiodensity. Accordingly, positive ΔSFD values reflect an increase in radiodensity (i.e., a shift toward less negative HU values), whereas negative ΔSFD values indicate a decrease in radiodensity. For percentage-based delta parameters (ΔPMI, ΔSMI, and ΔPNI), the “low” category was defined as a change equal to or below the respective cut-off value, reflecting tissue loss or insufficient increase during treatment. For ΔSFD, the “low” category represented patients with stable or increased subcutaneous fat radiodensity over time (i.e., minimal decrease or a positive change in HU values), corresponding to insufficient or unfavorable subcutaneous adipose tissue remodeling during treatment. Receiver operating characteristic (ROC) analyses were performed using vital status (death) as the classification outcome to identify thresholds for risk stratification, rather than for time-dependent predictive modeling. Body composition and nutritional parameters were evaluated by dividing them into binary categories in survival analyses. Optimal cut-off values for baseline PMI (5.5 cm^2^/m^2^), baseline SFD (−35.45 HU), ΔPMI (12%), ΔSMI (10%), and ΔPNI (6.63%) were determined using ROC analyses on overall survival, based on the maximal Youden index. ROC analyses were used for threshold identification to facilitate risk stratification rather than for predictive modeling, as the discriminatory performance of baseline parameters was modest. These variables were divided into “low” and “high” groups, with values above and below the cut-off values. For baseline IMAC and SMI, as well as ΔSFD and ΔIMAC, ROC analyses did not yield clinically robust or statistically reliable thresholds; therefore, the median values were used for dichotomization in these parameters. Dichotomization was primarily used to facilitate clinical interpretability and group-based survival visualization. However, we acknowledge that cohort-derived cut-offs may increase overfitting risk and reduce generalizability; therefore, these thresholds should be validated in external cohorts. Thus, all baseline and change parameters were consistently analyzed as binary categories in survival analyses.

### 2.4. Endpoints

The primary endpoint of this study was overall survival (OS). OS was defined as the time from the start of nivolumab treatment to the date of death from any cause; patients alive were censored at the date of last follow-up. Secondary endpoints included progression-free survival (PFS) as the time from the start of treatment to the date of radiological progression or, in patients without progression, to the date of last seen or death. Treatment response, assessed according to PERCIST criteria, included objective response rate (ORR) (complete response) + partial response (PR) and disease control rate (DCR): CR + PR + stable disease (SD). Furthermore, the prognostic impact of body composition parameters (PMI, SMI, IMAC, SFD) at the start of treatment and their changes calculated at 3 months during treatment were evaluated. Survival outcomes stratified by sarcopenia status were also analyzed.

### 2.5. Statistical Analysis

Statistical analyses were performed using IBM SPSS Statistics software (version 25; IBM Corp., Armonk, NY, USA). Categorical variables were reported as numbers and percentages, and continuous variables were reported as medians and IQR (Q1–Q3) as appropriate for the distribution. Differences between categorical variables were compared using Pearson’s chi-square test or Fisher’s exact test, when appropriate, and continuous variables were compared using the Mann–Whitney U test. OS and PFS were calculated using the Kaplan–Meier method, and between-group differences were assessed using the log-rank test. Univariate Cox regression analysis was performed for factors affecting OS. Univariate Cox proportional hazards regression analyses were performed to identify factors associated with overall survival. To limit model complexity and reduce the risk of overfitting, variables were selected for inclusion in the multivariable Cox regression model based on univariate associations (*p* < 0.10) and clinical relevance. Although several body composition parameters reflect related biological constructs, they were evaluated together in multivariable analysis to explore their independent prognostic contributions, with cautious interpretation of results. Given the exploratory nature of the study and the limited sample size, we focused on parsimonious multivariable modeling. Proportional hazards assumptions were assessed visually using log-minus-log plots, and no major violations were observed. HR and 95% confidence intervals were reported for all variables. A two-sided *p* < 0.05 was considered statistically significant in all tests.

## 3. Results

### 3.1. Patient Characteristics

A total of 34.1% (*n* = 30) of 88 metastatic NSCLC patients were classified as sarcopenic. Demographic, clinical, and body composition characteristics of the patients are summarized in Table 1. Sarcopenic patients were significantly older (65 [60–69] vs. 61 [58–63] years, *p* = 0.004). There were no significant differences between the groups in terms of gender, smoking status, ECOG performance, histology, PD-L1 status, and metastatic sites. However, the presence of ≥4 metastases was higher in sarcopenic patients (*p* = 0.018).

Body composition analyses revealed that PMI and SMI values were significantly lower in sarcopenic patients, while IMAC and SFD values were significantly different (*p* < 0.05 for all). While no difference was observed in terms of ΔSFD and ΔPMI in dynamic changes, ΔPNI was significantly higher in the non-sarcopenic group (*p* = 0.012). In ROC analyses, baseline body composition parameters demonstrated limited discriminatory ability for overall survival (baseline PMI: AUC = 0.61, 95% CI: 0.46–0.75; baseline SFD: AUC = 0.61, 95% CI: 0.47–0.76). In contrast, treatment-related changes showed improved discrimination, particularly ΔSMI (AUC = 0.67, 95% CI: 0.54–0.81). Corresponding Youden index–derived cut-off values were applied for subsequent risk stratification in survival analyses.

### 3.2. Treatment Response and Survival Outcomes

**Figure 2:**
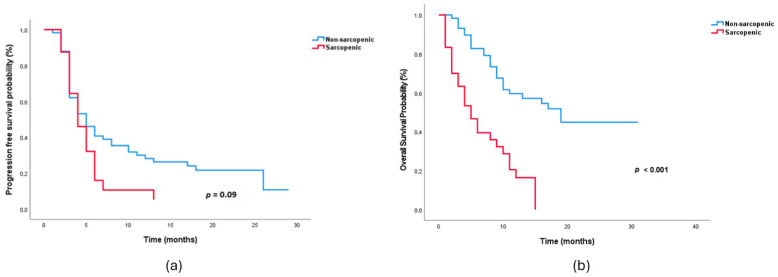
Kaplan–Meier curves for (**a**) progression-free survival and (**b**) overall survival according to baseline sarcopenia status.

Treatment response rates are shown in Table 2. The objective response rate (CR + PR) did not differ between sarcopenic and non-sarcopenic patients (33.3% vs. 53.4%, *p* = 0.073). The disease control rate was also similar between the two groups (*p* = 0.221). The median follow-up time was 18.0 months (95% CI: 15.8–20.2), as calculated using the reverse Kaplan–Meier method. Although PFS in sarcopenic patients was shorter than in non-sarcopenic patients, the difference was not statistically significant (median PFS: 4 vs. 5 months; log-rank *p* = 0.09). OS was significantly longer in the non-sarcopenic group (median OS: 19 months vs. 5 months; log-rank *p* < 0.001) (Figure 2a,b).

**Figure 3:**
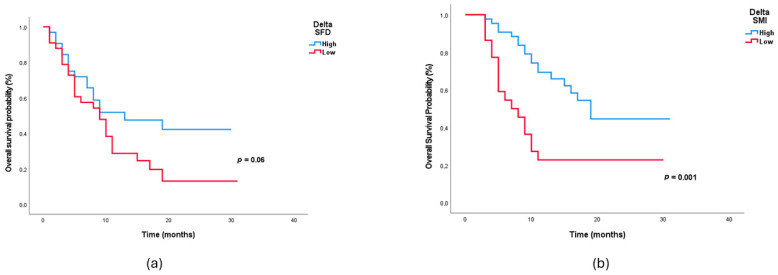
Overall survival according to (**a**) ΔSFD groups and (**b**) ΔSMI groups. *p*-values shown in the Kaplan–Meier curves are derived from log-rank tests and may differ from *p*-values obtained in Cox proportional hazards regression analyses.

In univariate analysis, age > 65, CCI ≥ 4, liver metastasis, low PMI, presence of sarcopenia, decreased ΔPMI, decreased ΔSMI, and decreased ΔPNI were found to be associated with survival. These results are presented in Table 3. In multivariate Cox analysis, only decreased ΔSMI (HR 3.39, *p* = 0.003) and unfavorable changes in subcutaneous fat radiodensity (ΔSFD (HR 2.45, *p* = 0.02) remained independent prognostic factors. Patients in the low ΔSFD group exhibited stable or increased subcutaneous fat radiodensity over time, corresponding to minimal decrease or a positive change in Hounsfield unit values. This pattern reflects the absence of favorable subcutaneous adipose tissue remodeling during nivolumab therapy. The effects of these two changes on survival are shown by Kaplan–Meier curves in Figure 3a,b. With respect to dynamic adipose tissue parameters, patients in the low ΔSFD group exhibited limited or insufficient change in subcutaneous fat radiodensity over time, suggesting the absence of favorable adipose tissue remodeling during nivolumab therapy. Interaction analyses were performed to evaluate whether the prognostic impact of treatment-related body composition changes differed according to PD-L1 status. No significant interaction was observed between PD-L1 expression and ΔSMI (*p* for interaction = 0.685) or ΔSFD (*p* for interaction = 0.131), indicating that the adverse prognostic effects of skeletal muscle and subcutaneous fat radiodensity remodeling during treatment were consistent across PD-L1 subgroups.

In general, the results show that muscle loss or insufficient gain and unfavorable change in subcutaneous fat radiodensity during treatment, are stronger predictors of survival than pre-treatment body composition.

## 4. Discussion

This retrospective study comprehensively evaluated the effects of both baseline body composition measurements and dynamic changes during treatment on survival in patients with metastatic NSCLC treated with nivolumab. The most striking finding of our study was that, although baseline sarcopenia was associated with significantly shorter survival in univariate analysis, it lost its significance as an independent prognostic marker in multivariate analysis. In contrast, the reduction in skeletal muscle mass (ΔSMI) and the unfavorable change in subcutaneous fat density during treatment were independently significantly associated with OS. These results suggest that compositional changes during treatment, particularly muscle loss, rather than static measurements, are the primary determinants of disease progression.

The impact of sarcopenia on immunotherapy outcomes has been extensively studied in recent years. Many studies report that baseline sarcopenia is associated with poor survival [14,15]. However, some of the literature, especially studies based on real-life data, does not support the independence of sarcopenia [16]. In our study, baseline sarcopenia was associated with OS in univariate analysis but lost significance in the multivariate model. This suggests that baseline sarcopenia acts as a “concomitant condition” because it is often associated with high metastatic burden, poor performance status, or other biologic components of cachexia, and may not be an independent predictor on its own. In addition, SMI-based sarcopenia definitions rely on a static anatomical threshold and may not fully capture the contemporaneous systemic inflammatory state or active cachexia burden at treatment initiation; therefore, its prognostic effect may be attenuated after adjustment for comorbidity and metastatic burden in multivariable models.

Dynamic assessment of muscle loss, however, carries much stronger prognostic value. Systemic inflammation, tumor metabolism, and immune dysregulation accelerate muscle breakdown, which can negatively impact the immune response [17,18]. Muscle loss, particularly during immunotherapy, may be directly related to the effectiveness of the treatment. In our study, skeletal muscle loss was found to be a strong and independent risk factor for OS. This finding is consistent with results in the literature. Muscle mass lost during treatment has been reported to be a much stronger prognostic parameter than baseline muscle mass [19].

Our findings on adipose tissue are also important. The fact that a unfavorable changes in fat density remained an independent risk factor in the multivariate model suggests the strong relationship between the fat compartment and inflammation, metabolism, and immune response. While the relationship between visceral fat and immunotherapy response has been studied more extensively in the literature, it is suggested that the subcutaneous fat radiodensity may be more closely related to immune reserve and metabolic resistance. The literature has shown that low subcutaneous fat is an independent predictor of mortality in cancer patients [20,21]. Our study supports that these relationships may also be valid in NSCLC patients receiving immunotherapy. Importantly, interaction analyses demonstrated no significant effect modification by PD-L1 expression for either ΔSMI or ΔSFD, suggesting that the adverse prognostic impact of treatment-related skeletal muscle and unfavorable change in subcutaneous fat radiodensity is consistent across PD-L1 subgroups. From a clinical perspective, early identification of these dynamic body composition changes may allow timely supportive interventions, such as nutritional counseling and individualized exercise strategies, potentially improving treatment tolerance during immunotherapy. From a biological standpoint, progressive skeletal muscle loss during immune checkpoint inhibition may reflect an amplified cachexia–inflammation axis and impaired immune competence. Chronic systemic inflammation (e.g., IL-6, TNF-α–driven pathways) promotes proteolysis and metabolic derangements, and sarcopenia has been linked to dysregulated immune cell function and reduced antitumor immunity. In this context, longitudinal muscle depletion may serve as an “integrative” marker capturing both tumor aggressiveness and host vulnerability during therapy, potentially contributing to reduced treatment tolerance and diminished immunotherapy efficacy [22].

The prognostic relevance of unfavorable change in subcutaneous fat radiodensity may also be biologically plausible. Subcutaneous adipose tissue (SAT) is not merely an energy depot but an active endocrine and immune organ producing adipokines and cytokines, and cachexia-related inflammatory signals can differ between adipose depots. In cachectic cancer patients, inflammatory alterations (including increased IL-6 expression) have been reported more prominently on SAT than in visceral adipose tissue (VAT), supporting the concept of depot-specific immunometabolic remodeling. Therefore, early radiodensity remodeling during nivolumab therapy may indicate loss of metabolic reserve and a shift toward a catabolic, pro-inflammatory state that compromises host resilience and may attenuate effective antitumor immune responses [23].

Another notable finding is that although both ΔPNI and baseline PNI were significant in the univariate analysis, their prognostic impact diminished in the multivariate model. While PNI reflects nutritional and immune reserve, it is highly sensitive to tumor burden and treatment-related fluctuations, which may limit its ability to function as an independent prognostic marker [24]. In this context, dynamic body composition parameters may better capture the ongoing catabolic and inflammatory state during immunotherapy than composite laboratory-based indices such as PNI.

This study has several notable strengths. First, it was conducted in a homogeneous cohort of patients with metastatic NSCLC, all treated with the same immune checkpoint inhibitor (nivolumab), which reduces treatment-related heterogeneity and enhances the interpretability of treatment-related body composition changes. Second, the single-center design ensured uniform clinical management, imaging protocols, and body composition assessment methodology, minimizing inter-institutional variability. Third, the study simultaneously evaluated dynamic changes in both skeletal muscle and subcutaneous adipose tissue during treatment, rather than relying solely on baseline measurements, providing a more comprehensive and clinically relevant assessment of host-related prognostic factors. Finally, the use of real-world data reflects routine clinical practice and supports the applicability of the findings to everyday oncology settings.

However, this study has several limitations that should be acknowledged. Its retrospective and single-center design, along with the relatively limited sample size, may restrict the generalizability of the findings. In addition, longitudinal body composition changes were assessed using PET/CT performed at 3 months, resulting in a landmark-type analysis that excluded patients with very early progression or death; therefore, the prognostic impact of ΔSMI and ΔSFD should be interpreted within the context of patients who remained on treatment beyond the initial 3 months. Furthermore, formal inter- and intra-observer variability analyses for body composition and HU-based radiodensity measurements were not performed due to the retrospective design, which should be considered when interpreting the reproducibility of ROI-based assessments. Treatment response was evaluated using PET-based PERCIST criteria rather than immune-modified morphologic criteria, and this should be taken into account when interpreting progression-related outcomes. Finally, the use of cohort-derived cut-off values based on ROC analyses may increase the risk of overfitting and warrants validation in external cohorts. In conclusion, our findings demonstrate that dynamic changes in body composition during immunotherapy, particularly muscle loss or insufficient gain and unfavorable changes in subcutaneous fat density, are much stronger prognostic indicators than baseline measurements. This finding highlights the importance of regular body composition monitoring throughout the treatment period, rather than clinical assessment based solely on baseline CT measurements.

## Figures and Tables

**Table 1:** Baseline demographic, clinical, and body composition characteristics according to sarcopenia status.

Variable	Non-Sarcopenic (*n* = 58)	Sarcopenic (*n* = 30)	*p* Value
Age, years	61 (58–63)	65 (60–69)	0.004
Gender, male	51 (87.9%)	27 (90.0%)	0.772
BMI, kg/m^2^	24.43 (22.15–28.19)	25.66 (22.77–29.75)	0.503
Smoking status			0.809
Current smoker	19 (36.5%)	11 (39.3%)	
Former smoker	33 (63.5%)	17 (60.7%)	
ECOG ≥ 1	28/55 (50.9%)	17/29 (58.6%)	0.500
CCI ≥ 4	35 (60.3%)	12 (40.0%)	0.070
Histology, non-squamous	35 (60.3%)	18 (60.0%)	0.975
PD-L1 negative	31 (60.8%)	12 (46.2%)	0.221
Treatment line (2nd vs. ≥3rd)	40 (69.0%) vs. 18 (31.0%)	23 (76.7%) vs. 7 (23.3%)	0.448
De novo metastasis	35 (62.5%)	19 (63.3%)	0.939
Number of metastases ≥ 4	21 (36.8%)	19 (63.3%)	0.018
Liver metastasis	8 (14.0%)	9 (30.0%)	0.074
Bone metastasis	24 (42.1%)	13 (43.3%)	0.912
Brain metastasis	13 (23.2%)	10 (33.3%)	0.312
Adrenal metastasis	12 (21.1%)	5 (16.7%)	0.624
PMI (baseline), cm^2^/m^2^	6.08 (5.26–6.99)	4.48 (4.06–5.83)	<0.001
SMI (baseline), cm^2^/m^2^	56.99 (52.34–62.73)	41.73 (37.90–48.20)	<0.001
IMAC (baseline)	−0.251 (−0.296−0.206)	−0.224 (−0.642–0.184)	0.048
SFD (baseline) HU	−36.7 (−40.0–−33.4)	−31.9 (−38.4–−25.0)	<0.001
ΔPMI	4.60 (−4.40–13.22)	17.53 (1.83–24.02)	0.250
ΔSMI	2.61 (−2.65–8.53)	12.22 (−9.72–28.95)	0.078
ΔIMAC	−3.28 (−36.99–19.69)	−0.29 (−51.95–−19.02)	0.905
ΔPNI	7.45 (−8.60–−14.29)	−6.65 (−16.31–6.62)	0.012
ΔSFD	−36.8 (−40.92–−32.7)	−34.4 (−39.1–−30.1)	0.278

Values are presented as n (%) for categorical variables and median (Q1–Q3) for continuous variables. Current smoker was defined as active cigarette smoking at the time of treatment initiation. Former smoker was defined as a history of smoking with cessation prior to treatment. Abbreviations: BMI, body mass index; ECOG, Eastern Cooperative Oncology Group; CCI, Charlson Comorbidity Index; PD-L1, programmed death-ligand 1; PMI, psoas muscle index; SMI, skeletal muscle index; IMAC, intramuscular adipose content; SFD, subcutaneous fat density; Δ, change from baseline to 3 months.

**Table 2:** Treatment response and survival outcomes according to sarcopenia status.

Variable	Non-Sarcopenic (*n* = 58)	Sarcopenic (*n* = 30)	*p*-Value
ORR, *n* (%)	31 (53.4%)	10 (33.3%)	0.073
DCR, *n* (%)	35 (60.3%)	14 (46.7%)	0.221
Median PFS, months (95% CI)	5.0 (3.2–6.8)	4.0 (2.7–5.3)	0.09
Median OS, months (95% CI)	19.0 (11.6–26.4)	5.0 (2.8–7.3)	<0.001

Abbreviations: ORR, objective response rate; DCR, disease control rate; PFS, progression-free survival; OS, overall survival.

**Table 3:** Univariable and Multivariable Cox Regression Analysis of Factors Associated with Overall Survival.

	Univariate Analysis			Multivariate Analysis		
	HR	95% CI	*p*	HR	95% CI	*p*
Age, >65	2.04	1.15–3.60	0.01	1.52	0.59–3.89	0.38
Gender, male	1.07	0.45–2.51	0.87			
Current smoker	1.00	0.55–1.82	0.98			
BMI ≥ 25	1.28	0.73–2.23	0.37			
CCI ≥ 4	2.23	1.27–3.90	0.005	2.11	0.96–4.59	0.06
ECOG ≥ 1	1.02	0.58–1.79	0.93			
Prior thoracic RT	1.22	0.70–2.12	0.47			
PD-L1 status < 1	1.54	0.86–2.76	0.14			
De novo metastasis	1.08	0.61–1.92	0.69			
Number of metastasis ≥ 4	1.20	0.69–2.07	0.50			
Liver metastasis	2.14	1.14–3.99	0.01	1.86	0.80–4.30	0.14
Bone metastasis	0.87	0.50–1.51	0.62			
Brain metastasis	1.48	0.82–2.65	0.18			
Adrenal metastasis	1.02	0.52–2.00	0.93			
PNI, low	1.68	0.97–2.92	0.06	1.71	0.81–3.59	0.15
PMI, low	2.11	1.21–3.67	0.008	1.02	0.38–2.70	0.96
SMI, low	1.35	0.78–2.33	0.28			
SFD, low	1.63	0.94–2.82	0.07	1.05	0.42–2.61	0.90
Sarcopenia, present	3.60	2.02–6.4	<0.001	1.09	0.41–2.88	0.85
IMAC, low	1.19	0.69–2.06	0.51			
ΔPMI, low	2.48	1.27–4.83	0.007	1.28	0.55–2.97	0.55
ΔIMAC, low	1.09	0.56–2.10	0.79			
ΔSMI, low	2.78	1.43–5.38	0.002	3.39	1.52–7.56	0.003
ΔPNI, low	2.52	1.39–4.58	0.002	1.94	0.83–4.50	0.12
ΔSFD, low	2.35	0.90–6.11	0.07	2.45	1.14–5.28	0.02

BMI: Body mass index, CCI: Charlson comorbidity index, ECOG: Eastern cooperative oncology group performance status, PD-L1: Programmed Death-Ligand 1, PNI: Prognostic nutritional index, PMI: Psoas muscle index, SMI: Skeletal muscle index, SFD: Subcutaneous fat density, IMAC: Intramuscular adipose content.

## Data Availability

Due to ethical and institutional restrictions regarding patient confidentiality, the datasets generated and analyzed in this study cannot be publicly shared. Anonymized data may be made available upon reasonable request with ethics committee approval.
